# Sensory white noise improves reading skills and memory recall in children with reading disability

**DOI:** 10.1002/brb3.2114

**Published:** 2021-06-06

**Authors:** Göran B. W. Söderlund, Jakob Åsberg Johnels, Bodil Rothén, Ellen Torstensson‐Hultberg, Andreas Magnusson, Linda Fälth

**Affiliations:** ^1^ Faculty of Teacher Education Arts and Sports Western Norway University of Applied Sciences Sogndal Norway; ^2^ Department of Education and Special Education University of Gothenburg Gothenburg Sweden; ^3^ Speech and Language Pathology Unit & the Gillberg Neuropsychiatry Centre Institute of Neuroscience and Physiology Silvia Children's Hospital University of Gothenburg & The Child Neuropsychiatric Clinic Gothenburg Sweden; ^4^ Department of Pedagogy and Learning Linnæus University Växjö Sweden; ^5^ Complex Adaptive Systems Chalmers University of Technology Gothenburg Sweden

**Keywords:** auditory white noise, dyslexia, noise benefit, reading disability, stochastic resonance, visual white pixel noise

## Abstract

**Background:**

Reading disability (RD) is characterized by slow and inaccurate word reading development, commonly reflecting underlying phonological problems. We have previously shown that exposure to white noise acutely improves cognitive performance in children with ADHD. The question addressed here is whether white noise exposure yields positive outcomes also for RD. There are theoretical reasons to expect such a possibility: a) RD and ADHD are two overlapping neurodevelopmental disorders and b) since prior research on white noise benefits has suggested that a central mechanism might be the phenomenon of stochastic resonance, then adding certain kinds of white noise might strengthen the signal‐to‐noise ratio during phonological processing and phoneme–grapheme mapping.

**Methods:**

The study was conducted with a group of 30 children with RD and phonological decoding difficulties and two comparison groups: one consisting of skilled readers (*n* = 22) and another of children with mild orthographic reading problems and age adequate phonological decoding (*n* = 30). White noise was presented experimentally in visual and auditory modalities, while the children performed tests of single word reading, orthographic word recognition, nonword reading, and memory recall.

**Results:**

For the first time, we show that visual and auditory white noise exposure improves some reading and memory capacities “on the fly” in children with RD and phonological decoding difficulties. By contrast, the comparison groups displayed either no benefit or a gradual decrease in performance with increasing noise. In interviews, we also found that the white noise exposure was tolerable or even preferred by many children.

**Conclusion:**

These novel findings suggest that poor readers with phonological decoding difficulties may be immediately helped by white noise during reading. Future research is needed to determine the robustness, mechanisms, and long‐term practical implications of the white noise benefits in children with reading disabilities.

## Graphical abstract

Here, we show experimentally that sensory white noise in two modalities improves reading and memory performance in children with relatively severe reading disability coupled with phonological (nonword) decoding difficulties. Moderate levels of visual white pixel noise improved both word reading and memory recall in children with phonological decoding difficulties. Auditory noise (80 dB) improved nonword reading in children with phonological decoding difficulties. Good readers and the children with poor orthographical word recognition but no phonological decoding difficulties performed worse in the visual noise condition and were not affected at all by auditory noise.

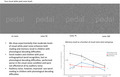

## 1 INTRODUCTION

Reading disability (RD) or dyslexia is among the most common neurodevelopmental disorders in children. The prevalence of RD commonly approximates 5%–12% of the population (Lyon et al., 2003; Peterson & Pennington, 2012). RD is characterized by significant difficulty in learning to read despite normal intelligence and sensory acuity and is known to have a considerable heritable basis (Peterson & Pennington, 2012). There is a broad consensus that an important proximal problem in word‐level reading disability relates to the ability to access and/or form stable phonological representations (Elbro & Petersen, 2004; Hulme & Snowling, 2009; Ramus et al., 2018) which negatively affects the ability to map the sounds of oral language to the letters of the alphabet. This phoneme–grapheme mapping difficulty is often assessed by nonword/pseudoword reading tasks. According to a recent integrative theoretical account of RD (Hancock et al., 2017), an increased rate of random fluctuations in background neuroelectric brain activity—often so‐called “neural noise”—may decrease the signal‐to‐noise ratios during information processing and thus weaken the associations between phonemes and graphemes in particular. Besides accounting for weaker phoneme–grapheme associations (Ahissar, 2007; Peterson et al., 2013) and, by extension, slow reading development, increased neural noise could potentially also explain other features of RD.

Reading ability is a strong predictor of lifelong school achievements (Nordström et al., 2016; Savolainen et al., 2008), and poor reading development is linked to mental health problems (Morgan et al., 2012). Hence, it is of crucial interest to develop tools and interventions for children that struggle with reading. One body of work has focused on trying to ameliorate impaired abilities in RD. In particular, children with RD have been shown to benefit from teaching approaches that directly and intensively target learning phoneme–grapheme relationships, so‐called phonics instruction (Bus & van IJzendoorn, 1999; Galuschka et al., 2014). While it is important that such instruction is provided to poor readers, it has also been noted that not all children respond well to phonics intervention (Torgesen, 2000). Research on individual response to intervention has suggested that impaired nonword reading is an important risk factor of poor response to phonics intervention (van der Kleij et al., 2017). That is, among poor readers, those who present with phonological decoding difficulties are less easily remediated through evidence‐based instruction. According to some prominent researchers (e.g., Siegel, 1989), poor pseudoword reading is in fact the clearest indicator of RD in children.

The other broad family of intervention measures relates to compensation. Technological advances, such as text‐to‐speech application, allow for access to text by circumventing the reading problem, for example, by “reading with the ears” (Edyburn, 2015). In school, another common adjustment is to provide a calm learning environment where poor readers can learn and practice reading without too much external noise and other distractions (Martinez, 2016). The latter kinds of adjustments might be even more important considering that RD has been linked experimentally with certain perceptual difficulties, such as speech perception difficulties against a background of other speech (Dole et al., 2014). Also, attentional difficulties are very common among children with RD (Hulme & Snowling, 2009). Indeed, findings show that substantial overlap exists between RD and ADHD, with published figures showing a comorbidity between 20% and 40% for these groups depending on the criteria applied (Mueller & Tomblin, 2012). This large overlap between the disorders provide, in our view, a clear indication that they may have some shared etiology, and indeed, the overlap between RD and ADHD has been shown on several levels of analyses, that is, behavioral and cognitive (Pennington, 2006), etiological (Willcutt et al., 2010), and genetic (Gialluisi et al., 2019). Among typical ADHD symptoms, RD displays a much stronger phenotypic and genotypic association with inattention than with hyperactivity‐impulsivity (Greven et al., 2011).

Recent research has suggested that the association between external noise, attention problems, and cognitive performance is quite complex (Pickens et al., 2019). External noise is typically thought of as a disturbance during cognitive activity, and humans often go to great lengths to reduce and avoid it. In essence, attention is a selection process that helps us focus on certain aspects of the world while filtering out others (Posner & Petersen, 1990). Signaling in the brain is thus characterized by noisy inputs and outputs, and an important task of the central nervous system is to distinguish the target signal, the information‐carrying component, from the surrounding noise, that is, meaningless neural input that interferes with the signal. Following exposure to unpredictable and uncontrollable high‐intensity noise, such as traffic or screams, the quality of cognitive task performance declines. Also, children in larger and acoustically noisy classes tend to show lower academic achievement, including poor reading performance (Earthman, 2002). From this perspective, it might seem counterintuitive that external noise, under some circumstances and for some individuals, can actually improve cognitive performance.

Indeed, there is today compelling evidence for the *benefits* of certain kinds of external auditory noise on cognitive performance in various tasks in children with attention deficits and/or an ADHD diagnosis (Helps et al., 2014; Söderlund et al., 2007). Research from our group has shown that attention and memory performance can be improved through auditory white noise exposure to inattentive and ADHD diagnosed children. For instance, in one study, it was found that exposure to white noise resulted in larger cognitive test improvement than from stimulant medication (Söderlund et al., 2016). The finding that white noise benefits cognitive performance has been replicated several times for different tasks and under different conditions, in children with an ADHD diagnosis (Baijot et al., 2016; Söderlund et al., ,,^2007^, ^2016^) and in teacher‐rated inattentive school children (Helps et al., 2014; Söderlund & Nilsson Jobs, 2016; Söderlund & Sikström, 2012; Söderlund et al., 2010).

The current study examines, for the first time, external sensory noise benefits in children primarily identified with RD. We see two main motivations for conducting such a study. *First*, it seems possible to invoke potential RD‐specific motivations. In particular, *the neural noise theory* proposes that information processing in RD is characterized by a low signal‐to‐noise ratio, especially during phonological awareness and grapheme–phoneme mapping, which has detrimental consequences for reading development in children with RD (Hancock et al., 2017). *Second*, the study is motivated by prior research on the benefits of white noise in inattentive/ADHD children, and the established overlap between RD and inattention symptoms.

Interestingly, Hancock et al., (2017) mention the phenomenon of stochastic resonance without explicitly suggesting the potential of manipulating noise levels. Doing so is therefore an important feature of the present study. Since *external* white noise has been shown to increase the signal‐to‐noise ratio through the phenomenon of stochastic resonance, details of which are further specified below, it might be expected that children with RD would display benefits from external noise. Further, direct or indirect support for the neural noise hypothesis in RD is found in brain imaging studies of altered neural variability, including functional magnetic resonance imaging (fMRI), in auditory brain stem responses (ABR), and in electroencephalography (EEG). In one study, using fMRI data, it was on the one hand shown that intraindividual neural variability or moment‐to‐moment changes in the reading network directly correlated with reading skills and that increased levels of BOLD signal variability aligned with better reading ability (Malins et al., 2018). On the other hand, by contrast, a study using auditory brain stem responses (ABR), poor readers showed significantly more variable brain stem responses to speech than good readers (Hornickel & Kraus, 2013). Thus, there are existing studies pinpointing the importance of neural variability in reading development and reading disability even though the direction and nature of the alterations might differ depending on the nature of the measures. Despite the just cited knowledge about neural noise relatively, few studies have experimentally introduced external sensory noise, in order to explore whether such an intervention affects the rather complicated interplay that exists between internal noise levels and task performance. Inspired by this view, the current study examines the influence of external white noise exposure on reading and memory performance in children with different reading skills. Since there might be specific neural atypicalities linked to phonological decoding difficulties (e.g., Díaz et al., 2012) and since previous research has pointed to nonword decoding as a marker of poor response to phonics intervention (van der Kleij et al., 2017), we performed analyses separately for poor readers with and without phonological decoding difficulties. The main hypotheses of the present study were that participants with RD would benefit from white noise whereas good readers would perform worse during white noise exposure.

The exact mechanism behind white noise benefits is not yet fully understood. Our guiding hypothesis in previous as well as in the present study has been the framework of Moderate Brain Arousal (MBA) that takes the phenomenon of stochastic resonance (SR) into account (MBA; Sikström & Söderlund, 2007). The concept of SR attempts to explain the paradox that the brain seems to utilize meaningless white noise to differentiate the signal in the targeted stimuli from nontarget noise. In particular, white noise accordingly improves or increases the signal‐to‐noise ratio (McDonnell & Ward, 2011). SR only appears in threshold‐based systems such as the nervous system and is usually quantified by plotting signal detection as a function of white noise intensity. The SR effect appears highly sensitive to both the intensity of the signal and the noise level; this relationship is presumed to follow an inverted U‐curve function, where performance peaks at moderate white noise levels. This means that a moderate level of white noise is beneficial for performance. By contrast, too little noise does not add the power required to bring the signal over the threshold to elicit an action potential, whereas too much noise overpowers the signal, leading to a deterioration in attention and performance (Moss et al., 2004).

The novel proposal of the MBA model is that there are individual differences in the amount of noise that is optimal for different brains. From the MBA model, one can predict that white noise benefit only occurs when a nervous system is not working at its optimum. In line with this prediction, sensory noise benefit on motor control has been seen in the conditions of various clinical groups, including in Parkinson (Novak & Novak, 2006), in diabetes and stroke (Priplata et al., 2006), in aging (Priplata et al., 2003), and, most well established, in ADHD. Hence, the MBA model can be helpful to explain why inattentive children will benefit more from higher levels of white noise than attentive children, for whom such noise levels will have a disadvantageous effect on performance.

**FIGURE 1:**
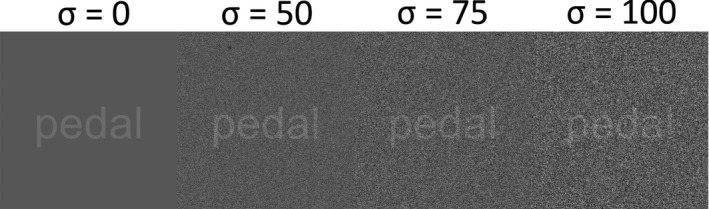
Shows images of the four visual white pixel noise levels used in the experiment *σ* = 0, 50, 75, and 100. *Note:* these word in noise examples are zoomed in on the words and do not show a full‐size video frame

The current study is the first to examine the effects of white noise exposure on word reading, orthographical lexical recognition, phonological (nonword) decoding, and word memory recall in children with and without RD. We also introduce white noise in the visual modality, using visual white pixel noise (Itzcovich et al., 2017) besides the auditory noise in our study to determine whether visual white noise might yield positive benefits as well (see Figure 1). In prior studies, cross‐modal effects have been shown whereby auditory white noise improved visuospatial tasks (Helps et al., 2014; Söderlund et al., 2016) and executive functioning (Baijot et al., 2016). Hence, it is not very far‐fetched to expect that similar cross‐modal benefits may be induced by visual white noise. If, as we hypothesize, white noise improves reading skills “on the fly” for poor readers, then this broadens the classroom support for a large proportion of children who struggle in schools today.

## 2 MATERIAL AND METHODS

### 2.1 Participant recruitment and screening

Ethical approval was obtained from the Ethical Review Board in Lund (EPN 2017/415; additional 2018/277). Written consent was obtained from the headmasters of three participating schools and from parents of participating children. All participating children gave oral consent to participate.

Following a screening phase in three schools in one region of Sweden, we recruited two groups of 10‐ to 13‐year‐old children defined by differing reading abilities: children with RD with phonologic decoding (nonword reading) problems, good readers, and poor readers with mild reading problems and no phonological decoding problems. All participants in the present study were recruited from the Swedish ordinary compulsory school where children are within the broad normal range of IQ (> 70). Regarding ethnicity and demographic sources, the three participating schools were in smaller towns or municipalities in mid and west Sweden with 3–30 000 inhabitants with similar demographic structure. Schools were also of comparable in size, 350–500 pupils in seven‐graded primary and midschools (children between 6 and 13 years). Participants with a foreign home language numbered roughly the same in the three participating schools, making up 20% of the total number, which is close to the average in Sweden. However, only children that are fluent in Swedish take part in the regular screening test, so that all screened children should have adequate command of Swedish.

The screening was conducted with the word‐chain test of orthographic word recognition ability (Jacobson, 2016). The screening included a total of 581 children in grades 4 to 6 (10–13 years) from the three schools. This is a standard screening procedure for all school children in the region, and it took place before the start of the present study (Jacobson & Lundberg, 2000). Altogether 82 school children participated in the experiments, and there were no dropouts from any of the groups. Children who received results corresponding to age‐adjusted stanine scores of 1–3 (23rd percentile, i.e., 23% out of the population) were assigned to either of the poor reading groups, whereas children with results corresponding to stanine 7–9 were assigned to the good reader group (controls). In order to create substantial differences between groups in reading performance, the rest of the children (i.e., those who scored in the midrange of stanine 4–6) were not included in the study. From the group that scored high in reading (7–9 stanine, a total of 68 children), 22 participants were chosen from the highest scores among the 68 on the list and assigned to the control group, named the *good readers*. Since the control group was chosen from the best readers (stanine 7–9), the variability within this group per definition will be low. This in turn suggests that additional participants would not add much power to the study, hence we decided to stop at 22 participants.

A total of 71 children with reading problems (stanine 1–3) were identified. Group assignment into the phonological/severe versus mild/orthographic poor reading groups was based on reading performance in the no‐noise condition on a subsequent individually administered test of phonological decoding, the nonword reading task from LÄST (Elwér et al., 2011). Children scoring low in phonological decoding (stanine 1–3), that is, they displayed poor performance in both the screening test of orthographical skills and the phonological decoding test, were assigned to one group, hereafter named the *phonologic or severe group* (*N* = 30). The rest, that is, children with at least average phonological decoding skills, but already assessed problems with orthographic performance in word chains, constituted the other group of poor readers, hereafter named the *orthographic or mild group* (*N* = 30). Poor reading participants were selected from lowest scores among the 71 poor readers children until we reached the prespecified number of 30 for each category (phonological/severe and orthographic/mild). Coincidently, the two groups added up to the target number of 30 in each group without the need to exclude anyone. Given the exploratory nature of the current work, the sample size for the two RD groups was based on power analyses from earlier experiences of noise research.

The group of RD with phonological decoding difficulties is our main group of interest for two reasons: this group is more challenged on all reading tasks, when compared with those that only have orthographical (lexical word recognition) difficulties and, moreover, several prominent researchers in the field have since long argued for the use of poor performance on a nonword reading test to define reading disability (e.g., Siegel, 1989). Hence, the phonological group might be the only group in relation to which it is meaningful to talk about RD at all. For more details about the participants, see Table 1.

**TABLE 1:** Participant characteristics: reading test scores and teacher ratings

Reading ability and teacher ratings	Phonologic grp Severe (Ph)	Orthographic grp Mild (Or)	Good Readers (GR)	Total (*N* = 82)
Boys / Girls	19/11	15/15	11/11	45/37
Age (Mean) Range	11.7 (10.2–12.8)	11.7 10.4–13.2	11.6 10.3–13.2	11.7 10.2–13.2
Grade 4 (≈ 10 years)	7	7	10	24
Grade 5 (≈ 11 years)	12	15	5	32
Grade 6 (≈ 12 years)	11	8	7	26
**Test scores**	**Mean (*SD*)**	**Mean (*SD*)**	**Mean (*SD*)**	
Word reading^a^	45.7 (12.0)	63.5 (10.0)	79.6 (12.4)	
Group comparisons				
Ph versus Or	*t*(58) = 6.26, *p* < .**001**			
Ph versus GR	*t*(50) = 9.92, *p* < .**001**			
Or versus GR	*t*(50) = 5.17, *p* < .**001**			
Nonword reading^b^	25.6 (6.4)	41.5 (6.7)	52.2 (7.4)	
Group comparisons				
Ph versus Or	*t*(58) = 9.43, *p* < .**001**			
Ph versus GR	*t*(50) = 13.9, *p* < .**001**			
Or versus GR	*t*(50) = 5.42, *p* < .**001**			
**Teacher ratings**	**Mean (*SD*)**	**Mean (*SD*)**	**Mean (*SD*)**	
Attention^c^ (SNAP score 0–27)	10.8 (6.0)	8.1 (7.7)	2.1 (3.5)	
Group comparisons				
Ph versus Or	*t*(58) = 1.55, *p *= .127			
Ph versus GR	*t*(50) = 6.06, *p* < .**001**			
Or versus GR	*t*(50) = 3.36, *p *= .**002**			
School achievement 1: below, 2: average, 3: above	1.5 (0.5)	1.7 (0.5)	2.8 (0.4)	
Group comparisons				
Ph versus Or	t(58) = 1.48, *p *= .143			
Ph versus GR	t(50) = 10.1, *p* < .**001**			
Or versus GR	t(50) = 8.28, *p* < .**001**			
Reading, writing ability 1: below, 2: average 3: above	1.1 (0.3)	1.6 (0.6)	2.8 (0.4)	
Group comparisons				
Ph versus Or	t(58) = 5.08, *p* < .**001**			
Ph versus GR	t(50) = 19.5, *p* < .**001**			
Or versus GR	t(50) = 8.53, *p* < .**001**			

^a^ Word reading, (Elwér et al., 2011) maximum score = 100.

^b^ nonword reading (Elwér et al., 2011). maximum score = 100.

^c^ SNAP score (Swanson et al., 2012), cutoff for ADHD‐I = 18p. All significant values are bolded ‐ maybe that is superfluous while both p‐values and t‐values are presented.

### 2.2 Teacher ratings of attention and school achievement

Due to the overlap between reading disability/ dyslexia and ADHD, all participants’ *attention abilities* were judged by their teachers, using the SNAP rating scale (Swanson et al., 2012). The SNAP score rates between 0 and 3 on attention ability. It consists of 18 questions that closely follow the DSM‐5 criteria for ADHD (APA, 2013). Nine questions assess attention ability and nine assess hyperactivity/ impulsivity. Here, only the attention scale was used. The 0 and 1 ratings are considered as normal scores, and the cutoff for ADHD‐I is a score of 18 or above. Regarding overall *school achievement,* teachers also rated the participants’ school performance on a three‐level scale (1–3) representing below average, average, and above‐average performance. Compared with the good readers, the two subgroups of poor readers displayed increased attentional problems according to SNAP scores and poor school achievement, while the poor reading groups did not differ significantly from one another in these regards. Note, however, that both RD groups scored, on average, well below the cutoff for a probable ADHD diagnosis. All figures and group comparisons are displayed in Table 1.

### 2.3 Procedure

All experiments were conducted at the participants’ schools, and participants were tested individually in a silent room alone with the researcher. Instructions were standardized, and the entire test session took approximately 30 min including instructions. The test order for the different tasks was the same for all participants. The order was as follows, *auditory noise tasks*: i) single word reading, ii) orthographical word recognition, and iii) nonword reading (decoding). Following this, the two *visual noise tasks were administered*: iv) single word reading, and v) verbal memory. In the auditory noise tasks, there were two trials, one for each of the two noise conditions (no noise versus noise) and the order of noise conditions was counterbalanced across subjects. In the *visual noise task,* four different noise levels were used, and the order of their appearance was also counterbalanced across subjects. Participants sat on a comfortable chair behind a desk where the word chains and nonword reading tasks were given on paper with and without auditory white noise.

#### 2.3.1 Visual white pixel noise

In the word reading and verbal memory task, all word stimuli in four visual noise conditions were presented on a computer screen using a 15” laptop. Children were seated approx. 70 cm away from the screen. The design of the visual noise was adapted after Itzcovich et al., (2017). The screen used has a resolution of 1,024 × 768 so in order to save rendering time but still having the same aspect ratio, the video clip size was set to 683 × 512 (i.e., ⅔ of 1,024 × 768). The clips’ frame rate was 20 frames/sec, and they were saved in the MP4 video format so that they could be played with a regular installation of a VLC media player. The colors in the video frames are limited to an 8‐bit grayscale color scheme with 0–255 ranging black to white. The background (BG) was set to 105 and the foreground (FG, the word) was set to 150, the numbers being a result of tuning with regard to visual perceivability. The noise is made by using random numbers according to *R* = *U* (−*σ*, *σ*) where *U* (−*σ*, *σ*) is the uniform distribution between –*σ* and *σ*. The values of *σ* are bound to |σ|≤105 since exceeding the BG color would fall outside the grayscale color scheme range. The noise is ultimately added to each video frame with an independent set of generated random numbers for each frame. The reason a Gaussian distribution was not chosen was to make the different noise levels as distinguished from one another as possible and this was more the case, appearance‐wise, using the uniform distribution (see Figure 1 for examples).

#### 2.3.2 Auditory white noise

In the auditory noise condition, the noise level was set to 80 dB in accordance with findings from earlier studies where positive noise effects were obtained (e.g., Söderlund et al., ,^2010^, ^2016^). In earlier research, several noise levels were tested in school children (Helps et al., 2014) and in a rat model of ADHD (Pålsson et al., 2011). The critical level for noise benefit in both studies was somewhere between 70 and 80 dB. From a more theoretical point of view, the literature describes two kinds of white noise facilitation: threshold SR and suprathreshold SR; we used the latter. These two types of noise benefits are differentiated by the nature of the relationship between the strength of the signal and the noise required for SR to occur (McDonnell et al., 2007; McDonnell & Ward, 2011). In auditory threshold SR, the signal should be presented just below the hearing threshold (20–35 dB, depending on age and frequency) and the noise should be within the same range (20–35 dB) for SR to occur, thus with a signal‐to‐noise ratio close to zero. In suprathreshold, SR will occur when all the noises being added are equal to the signal in terms of mean amplitude (McDonnell et al., 2007; Stocks, 2000). This means that both signal and noise can be far above the hearing threshold, in this case 80 dB, thus again, a signal‐to‐noise ratio close to zero. To induce cross‐modal SR, for example, effects of auditory stimulation on visual perception, suprathreshold levels are required within the same range, 70–80 dB (Manjarrez et al., 2007). Noise was delivered binaurally through high‐quality headphones (LOGITECH G433 7.1 Surround Headset) from a laptop computer.

### 2.4 Test battery and materials

#### 2.4.1 Single word reading

Single word reading was assessed by a Swedish translation of the word subtest from the Test of Word Reading Efficiency (TOWRE). The Swedish adaptation of this task is named LÄST, and hereafter, the test name LÄST will be used (Elwér et al., 2011; Torgesen et al., 1999). The participants were asked to read single words (on paper) aloud as fast as possible for 45 s (in each list). The test included two versions (A and B), the results of which were added up. The same test versions were used in noise and no‐noise condition, and the order of noise conditions was counterbalanced across participants. A test–retest reliability of 0.97 is reported in the manual (Elwér et al., 2011), and the total maximum score is 100.

#### 2.4.2 Orthographical word recognition (word chains)

Orthographic word recognition was assessed using the Word‐chain test (Jacobson, 2016). The task for the children was to silently read chains of words (on paper), where the blank space between words had been removed, and then mark each word boundary with a pencil. The task was to identify as many words as possible in *two minutes* (in one of two lists); one point for every correctly marked word was given. Each chain consisted of three semantically unrelated words. The two lists of word chains were counterbalanced over noise conditions across participants. Test–retest correlations for the Word‐chain test at a 12‐month interval range from *r *= .80 to .90 in different groups of children in Grades 1–6 (Jacobson, 2016).

#### 2.4.3 Nonword reading (phonological decoding)

Here, the participants were asked to read as many nonwords (on paper) as possible in 45 s from a list. The test included two versions (A and B), and the scores from the lists were added up. The same test versions were used in noise and no‐noise condition, and the order of noise conditions was counterbalanced across participants. The reported test–retest reliability for children aged 6–9 at this test was 0.97. The total maximum score is 100 (Elwér et al., 2011).

#### 2.4.4 Single word reading and word recall task (verbal memory)

This test consists of four video clips, one in each visual noise level, with 12 words displayed on a 15' computer screen. In each video clip, each word was exposed for 3 s, a fixation point (+) appeared at the center of the screen for 2 s as a fixation point between each word. The interstimulus interval was 5 s making a total of 60 s for the 12 words. The words were chosen as follows: six of the words were low‐frequency words, and six were high‐frequency words. Each word list was matched for word frequency, word length, and number of syllables. Participants read out each word aloud as they appeared on the screen. Prior to the presentation of the 12 words, used in the word reading assessment, participants were instructed to recall the words in any order they wished (free recall) after they finished the reading task. In order to prevent carry‐over effects, there were in total four different lists of words used in the experiment; they were counterbalanced so that the participants saw each word list once, and each word list appeared equally as many times in each noise condition across all participants. The order of visual white noise levels was counter‐balanced in the same way. The noise level measures were *σ* = 0, 50, 75, or 100 after Itzcovich et al., (2017).

## 3 RESULTS

### 3.1 Visual white pixel noise manipulation

**FIGURE 2:**
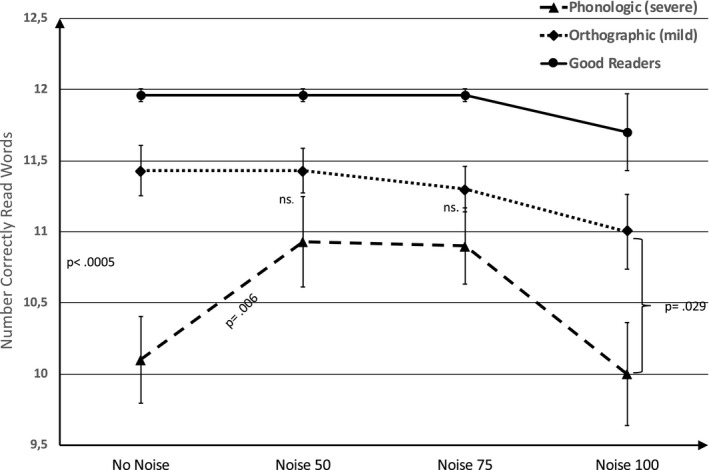
Number correctly read words as a function of visual noise level and group. *Note:* Error bars represent standard error of the mean

To explore the hypothesized effect of visual white noise, two separate two‐way mixed ANOVAs were conducted, one for the *word reading* task and one for the *word recall* task. There was a clear ceiling effect on the word reading task for the good readers. In order to avoid non‐normally distributed data, the first two‐way repeated measures ANOVA was carried out on the *word reading* task excluding the good readers, thus using a 2 × 4 design, with group as between subjects’ factor, group (2: phonologic /severe versus orthographic/mild) and one within subject factor, the visual white pixel noise condition (4: no noise, noise 50, noise 75, noise 100). Results showed two main effects: a main effect of visual white noise (*F* (3,176) = 5.735, *p* = .001, η^2^ = 0.090) and an effect of group (*F*(1,58) = 7.69, *p* = .007, η^2^ = 0.117), with the orthographic group reading words more correctly than the phonologic group. Importantly, there was an interaction between visual white noise and group that was curvilinear (quadratic) and driven by the performance of the phonologic/severe group whose reading performance improved during the two moderate visual noise levels (*F*(1,58) = 9.60, *p* = .003, η^2^ = 0.142) while the orthographic group performed worse in the visual noise conditions. When the same two‐way repeated ANOVA (3 × 4 design) was made including the good readers, results still showed a significant effect of visual white noise (*F*(3,156) = 5.55, *p* = .002, η^2^ = 0.178) and a main effect of group (*F*(2,79) = 13.36, *p *< .001, η^2^ = 0.253). The curvilinear interaction between noise and group remained significant (*F*(2,79) = 7.05, *p* = 002, η^2^ = 0.151). See Figure 2 for mean values and an illustration of the interaction between visual white noise levels. Post hoc tests of group comparisons, with Bonferroni corrections, showed that the good readers outperformed both the orthographic group (*p* = .006) and the phonologic group (*p* = < .001). A closer look at the differences between orthographic and phonologic groups at different noise levels, using independent samples *t* tests between the two groups for all noise levels were made separately (Bonferroni correction (0.05/4 = 0.0125 as criterium), showed that while the orthographic group outperformed the phonologic group in the no‐noise condition (*t*(58) = 3.78, *p *< .001), this only indicated a trend in the highest noise condition, that is, noise 100 (*t*(58) = 2.24, *p* = .029). In the two middle noise conditions (noise 50 and 75), the groups were not significantly different (N50: *t*(58) = 1.41, *p* = .164 and N75: *t*(58) = 1.28, *p* = .206). The last post hoc tests conducted were paired samples *t* tests within groups that showed that performance improved compared with no noise during visual noise at noise level 50 (*t*(29) = 2.98, *p* = .006, Cohen's d = 0.54), and at noise level 75 as well (*t*(29) = 3.13, *p* = .004, Cohen's d = 0.57) in the phonologic group only. When gender and age were inserted as covariates in the above ANOVA, this did not change data at all, neither main effect nor interactions.

**FIGURE 3:**
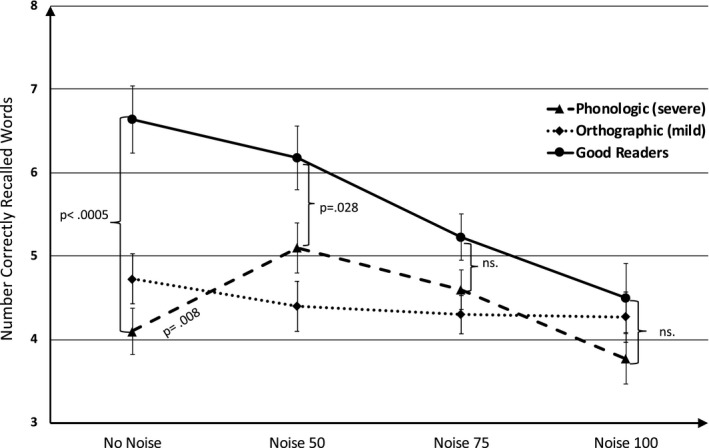
Number correctly recalled words as a function of visual noise level and group. *Note:* Error bars represent standard error of the mean

The second two‐way repeated measures ANOVA was carried out on the *word recall* task (a 3 × 4 design) with group as the between individuals’ factor (3: good readers versus orthographic versus phonologic group) and visual noise level as the within individual factor (4: no noise versus noise50 versus noise75 versus noise100). Again, two main effects were obtained, a negative main effect of visual white noise (*F*(3,77) = 10.64, *p *< .001, η^2^ = 0.119) and an effect of group (*F*(2,79) = 9.3, *p *< .001, η^2^ = 0.119). Post hoc tests across all conditions revealed Bonferroni‐corrected group differences such that good readers outperformed both the orthographic group and the phonologic group (*p *< .001 and *p* = .001, respectively), while the orthographic and phonologic groups did not differ significantly from each other (*p* = 1.0). Importantly, however, the interaction between visual white noise and group was significant (*F*(6,237) = 4.51, *p *< .001, η^2^ = 0.102). Again, the effect was curvilinear (quadratic) (*F*(2,79) = 4.88, *p* = .010, η^2^ = 0.110). Figure 3 illustrates mean values and the interaction between visual white noise levels and groups and how the phonological decoding difficulties group performed better under the two moderate noise levels while they performed worse under the no‐noise and high visual noise conditions. Paired samples *t* tests confirmed that the phonologic group performed better at noise level 50 (*t*(29) = 2.87, *p* = .008, Cohen's d = 0.52) compared with no noise. See Table S1 for all mean values and standard deviations for the two visual noise tasks. When gender and age were inserted as covariates in the above ANOVA, this did not change data at all, neither main effect nor interactions.

### 3.2 Auditory white noise manipulation

In order to evaluate the influence of auditory white noise, three separate two‐way repeated measures ANOVAs (3 x 2) were conducted for each of three reading tasks: single word reading, orthographical word recognition (word chains), and nonword (phonological) decoding. All three ANOVAs included group as the between subjects’ factor (good readers versus orthographic versus phonologic) and noise as a within subject factor (no noise versus white noise). The results for the *single word reading* task showed a main effect of group (*F*(2,79) = 53.35, *p *< .001, η^2^ 0.575) but no main effect of noise or interaction between group and white noise (*F* < 1). Post hoc test after Bonferroni corrections showed that the good readers outperformed the orthographic group, who in turn performed better than the phonologic group (both *p *< .001). Mean values and standard deviations are displayed in Table 2. When gender and age were inserted as covariates in the above ANOVA, this did not change data at all, neither main effects nor interactions.

**TABLE 2:** Participants test scores in word reading, orthographic word recognition, and nonword decoding tasks in auditory noise conditions

Task/Group	Word reading		Orthographic lexical task		NonWord reading	
Noise condition	No noise	Noise	No noise	Noise	No noise	Noise
Phonologic grp, severe						
*M* (*SD*)	45.7 (12.0)	47.4 (11.1)	24.1 (7.1))	25.6 (6.9)	25.6 (6.4)	28.9 (7.4)
Range	23–69	24 –63	12 –36	15–38	13–34	12–40
No noise versus Noise	*t*(29) = 2.26, *p *= .**032**		*t*(29) = 3.68, *p *= .**001**		*t*(29) = 5.32, *p* < .**001**	
Orthographic grp, mild						
*M* (*SD*)	63.5 (10.0)	64.3 (8.8)	27.3 (5.9)	28.1 (6.7)	41.5 (6.7)	41.4 (6.1)
Range	40–81	48–80	15–38	12–45	28–57	33–59
No noise versus Noise	*t*(29) = 1.01, *p *= .321		*t*(29) = 0.97, *p *= .340		*t*(29) = 0.08, *p *= .933	
Good readers						
*M* (*SD*)	79.6 (21.4)	78.9 (17.2)	43.8 (7.2)	44.6 (12.3)	52.2 (7.4)	52.7 (8.5)
Range	39–95	36–101	32–56	28–87	39–63	34–66
No noise versus Noise	*t*(21) = −0.25, *p *= .806		*t*(21) = 0.45, *p *= .660		*t*(21) = 0.81, *p *= .425	

The second two‐way repeated measures ANOVA (3 × 2) was conducted for the *orthographic word recognition* *task* (word chains). Results showed, again, a main effect of group (*F*(2,79) = 49,9, *p *< .001, η^2^ = 0.558) and a weak tendency to a main effect of white noise (*F*(1,79) = 3.20, *p* = .078) but no interaction between white noise and group (*F* (2,79) = 0.196, *p* = .822). Post hoc test after Bonferroni corrections showed that good readers performed better than both orthographic and phonologic group (both *p *< .001), but no difference was obtained between the orthographic and phonologic group (*p* = .390). See Table 2 for mean values and standard deviations. When gender and age were inserted as covariates in the above ANOVA, this did not change data at all, neither main effect nor interaction.

**FIGURE 4:**
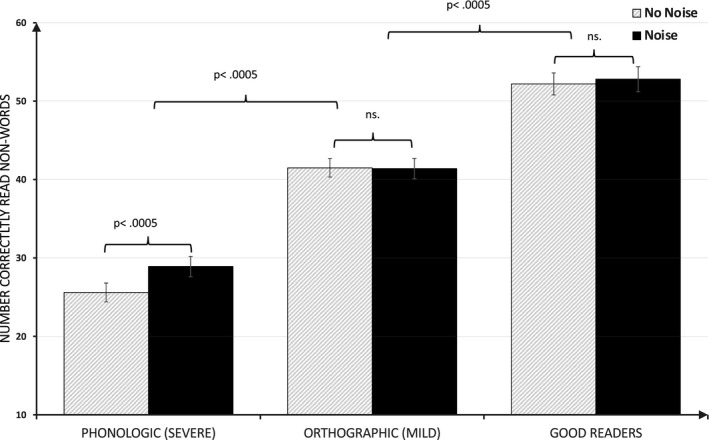
Number correctly read nonwords in 45 s as a function of auditory noise level and group. *Note:* Error bars represent standard error of the mean

The third two‐way repeated measures ANOVA (3 × 2) was conducted for the *nonword* *decoding* task. In this ANOVA, results showed two main effects: one of white noise (*F*(1,79) = 9.32, *p* = .003, η^2^ = 0.106) and the second of group (*F*(2,79) = 90.2, *p *> .001, η^2^ = 0.696). Post hoc tests after Bonferroni corrections showed that good readers performed better than the orthographic group (*p *< .001) who in turn outperformed the phonologic group (*p *< .001). Similar to the visual noise tasks, in this ANOVA, results showed a significant interaction between noise condition and group (*F*(2,79) = 6.90, *p* = .002 η^2^ = 0.149). As revealed in Figure 4, this interaction appeared to be driven by a distinctive white noise improvement in the phonologic group. Post hoc paired samples *t* tests confirmed a significant white noise effect in the phonologic group (*t*(29) = 5.32, *p *< .001, Cohen's *d* = 0.97) but no effect of noise neither for the good readers (*t*(21) = 0.814, *p* = .425) nor for the orthographic group (*t*(29) = 0.085, *p* = 933). All mean values and standard deviations for the three above tasks are found in Table 2 above. When gender and age were inserted as covariates in the above ANOVA, this did not change data at all, neither main effect nor interaction.

### 3.3 Dimensional analysis of the full sample

Group assignment in reading disability is somewhat problematic since it is commonly recognized that reading skills are dimensional along a continuum rather than binary in “good” versus “poor” reading. Yet, it is oftentimes meaningful to make groupings when doing comparisons between participants with different features such as reading skills, and this was indeed the approach taken in the main analyses in the study. To avoid uncertainties in this regard, however, a set of complementary correlation analyses were conducted for all outcome variables on the full data set (*N* = 82 participants) to examine whether the main results remained scores in dimensional analyses. A noise benefit variable was formed for all tasks in both visual and auditory noise conditions, by calculating the difference between the score in the noise condition minus the score in the no‐noise condition (i.e., the delta value). For the visual noise tasks, the moderate noise level with the largest effect size was chosen (noise *σ* = 50). The Pearson correlation showed a significant correlation between word reading in the noise free condition and noise benefits (delta) in the visual noise condition (noise *σ* = 50) (*r* = −.556, *p* < .001). In the word recall condition, the Pearson correlation between word recall and noise benefit in word recall was also significant (*r* = −.570, *p* < .001). To ensure that these noise effects were not driven by attention deficits, the effect of attention ability assessed with SNAP was partialled out; the correlations in both variables remained significant and almost identical in strength (*r* = −.545, *p* < .001 and *r* = −.564, *p* < .001, respectively).

The same procedure was followed for the three auditory noise tasks (word decoding, word recognition in word chains, nonword reading). The outcome variables, measured as noise benefits (delta) in the same three tasks for the entire group, revealed the following results: word reading performance and noise benefit in reading showed a nonsignificant marginal correlation (*r* = −.203, *p* = .068), the word‐chain performance and noise benefit were uncorrelated (*r* = −.014, *p* = .898) while the nonword reading scores significantly correlated with noise benefit in the same task (*r* = −.343, *p* = .002). Again, when the effect of attention ability assessed with SNAP was partialled out, the correlation with nonword reading remained significant (*r* = −.358, *p* = .001). Finally, these relative noise benefit (or nonbenefit) values were subjected to correlation analysis with attention scores as measured by the SNAP. Bivariate Pearson's correlation analyses were conducted with the entire sample of participants (*N* = 82); no significant correlations were found between attention ability and noise effects. Taken together, the above assessments provide evidence for the suggestion that noise benefits, in both visual and auditory domains, are robust in respect to these complementary dimensional analyses. Moreover, the benefits do not appear to be driven by attention deficits.

### 3.4 Participants’ experiences from noise exposure

In debriefing, all participants were asked their opinion about being exposed to white noise during the different tests. Two questions were asked with three alternatives in each noise condition: i) which auditory noise condition did you find most pleasant? With white noise; without white noise; or both worked? ii) which one of the visual white noise conditions did you find most pleasant? With some visual noise; with lots of visual noise; without visual noise. The two main findings emerged. *First*, there were no significant differences in noise preferences between groups. *Second*, a majority (≈70%) of all participants did not mind auditory white noise and just over 30% preferred a silent condition. Regarding visual white noise, the preference for noise was even stronger, over 90% preferred the noisy screen and only 7% preferred the noise free one. Finally, none of the 82 participants reported any adverse experience from the white noise exposure (see Table S2 for exact figures and Chi‐square values).

## 4 DISCUSSION

This proof‐of‐concept study is the first to provide evidence of the beneficial impact of sensory white noise on the reading and memory performances of children with reading disability (RD) associated with phonological (nonword) decoding problems. The positive white noise effects were present in two modalities, namely in visual and, less consistently, auditory modalities. With regard to visual white noise, an inverted U‐curve on both the reading and the memory recall tasks were obtained in the group with RD and phonological decoding deficits. This means that lowest performance was found in the zero and maximum visual white noise conditions, whereas the two middle visual white noise levels improved word reading and memory recall performance. In all, the current study provides promising results for improving the reading capacities “on the fly” in children with RD and phonological decoding difficulties.

Data depicting an inverted U‐curve are also of considerable theoretical interest since it is in line with predictions from the MBA model in which the phenomenon of stochastic resonance is presumed to play a key role. In the auditory condition, the poor readers with phonological decoding difficulties improved most clearly on the nonword reading task, in which this group had previously performed markedly weaker than the other two groups.

Another striking result was that the white noise affected the three participant study groups differently. Skilled readers displayed no improvements with auditory white noise and showed a similar pattern on the visual noise tests, in which the white noise seemed to exert a strong linear negative impact on performance. We will discuss this issue in greater detail below in light of possible theoretical implications for the Moderate Brain Arousal model and the putative stochastic resonance phenomenon (Sikström & Söderlund, 2007). From a practical point of view, we thus found a treatment*aptitude interaction which has very rarely been reported in the RD field more generally (Fletcher & Grigorenko, 2017) despite much speculation about the potential of targeted educational “treatments” for certain subgroups of school children. Noteworthy is the steep decline on word memory performance in the good readers group when white noise was introduced in the visual domain, which was evident despite the fact that they decoded the words accurately in the reading condition. This finding is in line with prior research showing that visual noise damages memory encoding of lexical items in skilled readers (Gao et al., 2011). Indeed, prior research on skilled readers have shown environmental noise more generally impairs the ability to instantiate word meanings and integrate them in text as revealed in behavioral and brain (e.g., in event‐related potentials) data (Aydelott et al., 2006).

The orthographic group—who presented with somewhat milder word reading problems and no difficulties with nonword (phonological) decoding—responded largely similar as the skilled readers on white noise exposure, although displaying lower performance overall. Future research is needed to detail why this group with poor reading did not respond positively to the noise manipulation. Given that the design and hypotheses of the current study were partly informed by prior research on children with ADHD/inattentive symptoms, it could perhaps be suggested that differences in such symptoms might explain group differences in response to the white noise manipulation. However, subclinical traits of inattention were found to be higher in both poor reading groups when compared with the skilled readers, while they did not differ from each other in this regard (see Table 1 in the method section); moreover, when controlling for inattentive traits in a dimensional analysis, the main conclusions remained (supplementary material). Instead, it might be worth considering that orthographic processing difficulties in prior reading research have been shown to associate more closely with measures of reading habits and limited print exposure (Cunningham & Stanovich, 1993). Hence, the orthographic group in our study may have included relatively more children who mainly struggle with reading due to limited reading experience rather than being caused by a disability, which might be the case in the phonologic group (cf. Siegel, 1989). The fact that the orthographic group had somewhat milder word reading problems is potentially also in line with such a suggestion.

Regarding the effect sizes of the noise benefits—which are obviously important from the sake of practical implications—we found Cohen's d values between 0.52 and 0.97; these effects are considered as being of medium up to large size according to established criteria. Moreover, these effect sizes are in parity with or even slightly larger than the ones found in ADHD research on white noise benefits: In word memory recall tasks, effect sizes (Cohen's d) have been found to be of medium size, between 0.41–0.46 (Söderlund et al., 2007, 2010), whereas in a visuospatial working memory task and in executive functioning tasks the effect sizes were large, d > 0.80 (Baijot et al., 2016; Helps et al., 2014; Söderlund et al., 2016). Thus, in ADHD, we have received larger noise effect sizes in executive functioning tasks as compared to memory tasks. Speculatively, it appears that the more difficult a task is for a given group, the larger is the white noise benefit. That hypothesis seems applicable also in the present data set in which there was a large noise benefit effect for nonword reading in the auditory noise experiment. We will explore these features in greater detail in future research.

### 4.1 Comparisons with earlier noise research in RD

The present findings may seem idiosyncratic compared to what is known from earlier research on perceptual deficits and increased vulnerability to noise exposure in RD, for instance while processing speech (Beattie et al., 2011; Sperling, Lu, Manis, & Seidenberg, 2005, 2006; Ziegler et al., 2009). In relation to the phenomenon of SR, the concept of noise is elusive, however. To actually yield a noise benefit in terms of signal detection, the exact properties of the noise seem to be of paramount importance: 1) the signal‐to‐noise ratio has to be exact, implying that several noise levels have to be used to identify the “right” noise level; 2) random, noninformation‐carrying noise seems to be necessary (McDonnell & Abbott, 2009; Sikström & Söderlund, 2007) whereas all other kinds of noises might interfere with the information‐carrying signal; 3) the power spectrum of the noise preferably should be flat or uniform for SR to occur, whereas this is less likely in Gaussian and/or speech shaped noise (Stocks, 2001; Zozor & Amblard, 2003). In prior research on noise perception in RD—including seminal studies by Sperling et al., (2005), Sperling et al., (2006) and Beattie et al., (2011)—children with RD generally manifested difficulties in excluding distractors and therefore performed worse in noisy conditions. However, none of the paradigms used were designed to elicit SR according to the conditions described above. Hence, we do not consider the current findings to be in conflict with any prior research on noise exclusion difficulties in RD. An important topic for future research is to precisely unravel when and what kinds of noise improve versus impair performance in children with RD.

### 4.2 Theoretical and practical considerations

Although tentative at this stage, there might be more specific neurobiological differences that can explain why only the “phonological” group responded favorably to white noise. We think the recent neural noise hypothesis of dyslexia provides a particularly compelling context for interpreting the results of the study, and indeed, the noise benefit framework developed here can potentially add to current understandings of RD, in particular when considered in relation to a proposed multi‐modal sensory information integration deficit. The neural noise model suggests that neural hyperexcitability will disrupt multisensory integration that is of crucial importance for phonological processing and for mapping between phonemes and letters (Hancock et al., 2017). It also seems possible from this account to predict that poor readers with and without phonological difficulties will differ in the amount of neural noise abnormality, which might help explain why only the phonologic group (who also were more severely affected) displayed external white noise benefits here. Moreover, the balance between inhibitory and excitatory activity in a neural network can be disrupted by excessive excitatory input leading to increased variability in neural firing and a loss of spike timing precision and thus a loss of neural network synchronization (Hancock et al., 2017). This random firing can be quantified as neural gain using a sigmoid function (Hauser et al., 2016). A key hypothesized feature of SR is that when internal noise levels are high, added external white noise via the sensory system (in any modality) produces a less random output, or in other words an increase of the signal‐to‐noise ratio. It is interesting to note that Hancock et al. (2017) in their landmark paper mention the phenomenon of SR while not explicitly considering the idea of manipulating external noise levels in order to investigate if dyslexics are responsive to white noise or the SR phenomenon as such. Our study contributes new knowledge in this regard. Although the evidence is clearly indirect, changes in neural variability are here proposed as one of the possible mechanisms behind the observed effects of external sensory noise in the present study.

There is now plenty of evidence in SR research that different neural systems require different amounts of external white noise (auditory, tactile, visual, vestibular) to work optimally, and, in particular, systems that are impaired to start with: we see greater noise benefit in the SR context (Kim et al., 2013; Ward et al., 2006). This notion appears to correspond well with proposals of inverted U‐shaped associations between increases and decreases in neural BOLD signal variability over the life span, leading to decreased behavioral performance (Nomi et al., 2017); or in trial to trial brain activity and behavioral output in electrocorticography (EEG) recordings (He & Zempel, 2013). Altogether this supports theoretical frameworks such as the MBA model that regards the brain as an active nonlinear dynamic system rather than a passive signal‐processing device (Sikström & Söderlund, 2007).

Of course, there are alternative explanations of noise benefits besides those implicated by the MBA model (i.e., stochastic resonance). A good candidate is auditory masking where noise screens out other possible distracting stimuli (Breier et al., 2002). In threshold SR, a weak near threshold stimulus, that is constituted by a masker different from the signal, can facilitate signal detection (Durlach et al., 2003). Moreover, masking effects have been shown in both visual (Dawes et al., 2009) and tactile modalities (Tan et al., 2003). Another explanation worth considering is that, rather than inducing SR, the white noise exposure increases physical arousal in participants which in turn will affect inattentive persons, that are suggested to be underaroused, different from attentive persons. Such an explanation is consistent with the state regulation model of ADHD (Sonuga‐Barke et al., 2010) derived from the cognitive energetic theory (Sergeant, 2005). However, very little is known about energetic or arousal levels in RD and dyslexia, although one study using resting state quantitative EEG found that children with combined ADHD and RD had more relative theta, less relative alpha, and a higher theta/alpha ratio in their EEG when compared to children with ADHD without reading difficulties (Clarke et al., 2002). These findings may indicate that individuals with RD display levels of underarousal in the nervous system that go over and above any comorbid attention disorder. The observation that patients with dyslexia have been treated with some success with medication targeting dopamine agonists (Keulers et al., 2007) and by atomoxetine that acts on the norepinephrine system (Shaywitz et al., 2017), provides further support for this suggestion. Future research is clearly needed in this area.

Regardless of the exact reason for the observed subgroup differences, it is noteworthy that prior intervention research on unselected samples of children with poor word reading has shown that children with poor phonological decoding ability often respond less well to state‐of‐the‐art phonics interventions aimed at ameliorating word reading skills (van der Kleij et al., 2017). Thus, white noise manipulation may provide external help to those that need it most. Besides independent replication of the results, the long‐term implications of practicing reading with white noise need to be explored in future research. There are, as we see it, three possible outcomes from such research. *The first* possibility is that the benefit of white noise for reading works instantly and is present only during the noise exposure, comparable to how a pair of reading glasses works. *The second* possibility is that long‐term reading training with noise yields permanent improvements in reading also when no white noise is present, that is, the benefits become permanent or crystallized. The latter would not be an unreasonable supposition, since accurate phonological decoding is often considered a “sine qua non” mechanism of fluent reading acquisition, for instance in Share's self‐teaching hypothesis (Share, 1995). *The third* possibility that has to be considered seriously is that long‐term use of noise may lead to habituation, with noise benefit fading after some time without securing neither short nor long‐term benefits in performance.

### 4.3 Limitations and future directions

There are a number of limitations in the current proof‐of‐concept study that need to be addressed in future research. Although we see it as a considerable strength that the experiments were conducted in schools with children identified through general screening of reading skills, practical considerations hindered us from conducting in depth clinical examinations of each child in this setting. Thus, we have no assessment information regarding factors such as family heritability, or a number of commonly assessed cognitive skills such as working memory, IQ, or rapid automatized naming. In particular, we do not know whether the groups differ in IQ, general cognitive, or perceptual ability, which might have affected the results. Future research is needed to examine whether IQ differences among poor readers affect the response to noise exposure. One further limitation is that we used the outcome variable, nonword reading task, as grouping variable. This was due to the lack of nonword reading tests in Swedish. However, we are reassured that the positive results of noise are not only present in the nonword reading but is approaching significance also in word chains (auditory noise) and, in particular, is significant both in word reading and word recall in visual noise. We consider the visual noise benefits as our most important findings. Another task‐related limitation is that we did not use the same outcomes in the auditory and the visual noise experiments, which prohibits comparisons of their relative effects. One important reason for not doing so is that the visual noise must be displayed on a computer screen, whereas most traditional assessments of reading are done with pen and pencil. Also, the visual noise word stimuli were embedded in videoclips (adapted after Itzcovich et al., 2017), and this technique is not suitable for showing many items or longer texts simultaneously on screen.

## 5 CONCLUSION

This study showed that the group of children with RD displaying phonological decoding difficulties responded differently to white noise exposure than the two other groups tested. The phonological group displayed white noise benefit in several of the tasks, whereas the two other groups showed a decline or no effect at all under the same levels of white noise exposure. This pattern of white noise benefit was present in both modalities, that is, in the visual and auditory noise conditions, although more evident during visual noise. Moreover, in the visual noise condition, the noise benefit was present both during reading and retrieving words (memory recall). We propose tentatively, in accordance with the MBA model, that these noise benefits are caused by the phenomenon of stochastic resonance in which weak signals can be amplified through task irrelevant sensory white noise. The results might be of both theoretical and practical importance for understanding and supporting children with RD.

## CONFLICTS OF INTEREST

None of the contributing authors have any conflicts of interest to report.

## AUTHOR CONTRIBUTIONS

G.S. and L.F. conceived and designed the experiments and wrote the first draft. BT, ETH, and LF performed experiments. G.S. analyzed data and made figures and tables. G.S. and J.Å.J. wrote the paper, revision and cover letter.

### PEER REVIEW

The peer review history for this article is available at https://publons.com/publon/10.1002/brb3.2114.

## Supporting information

Fig S1‐S2

Table S1‐S2

## Data Availability

Data available on request from corresponding author Göran Söderlund email: goran.soderlund@hvl.no.
