# Supplementary material for: Sensory white noise improves reading skills and memory recall in children with reading disability
Source: Brain Behav. 2021 Jun 6;11(7):e02114. doi: 10.1002/brb3.2114 (PMC8323032; doi:10.1002/brb3.2114)
Supplement: Supplementary file 1 — Fig S1‐S2 [file BRB3-11-e02114-s002.pdf]

Box plots visual noise

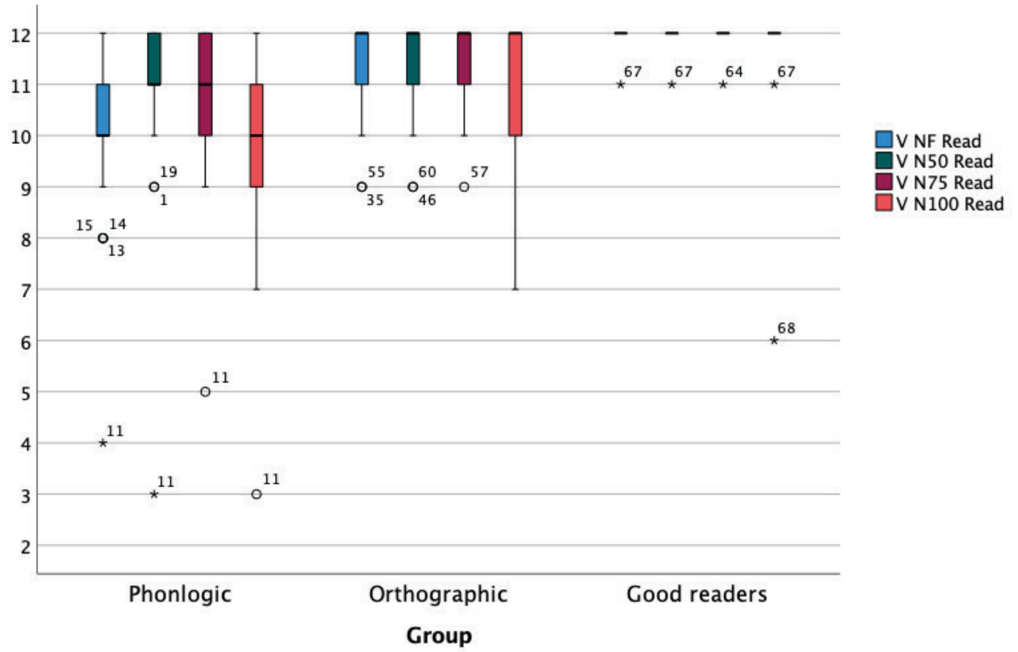

Figure 1. Boxplot showing word reading performance as a function of visual noise level (we did exclude the outlier (11) in the phonologic group and this did not change data)

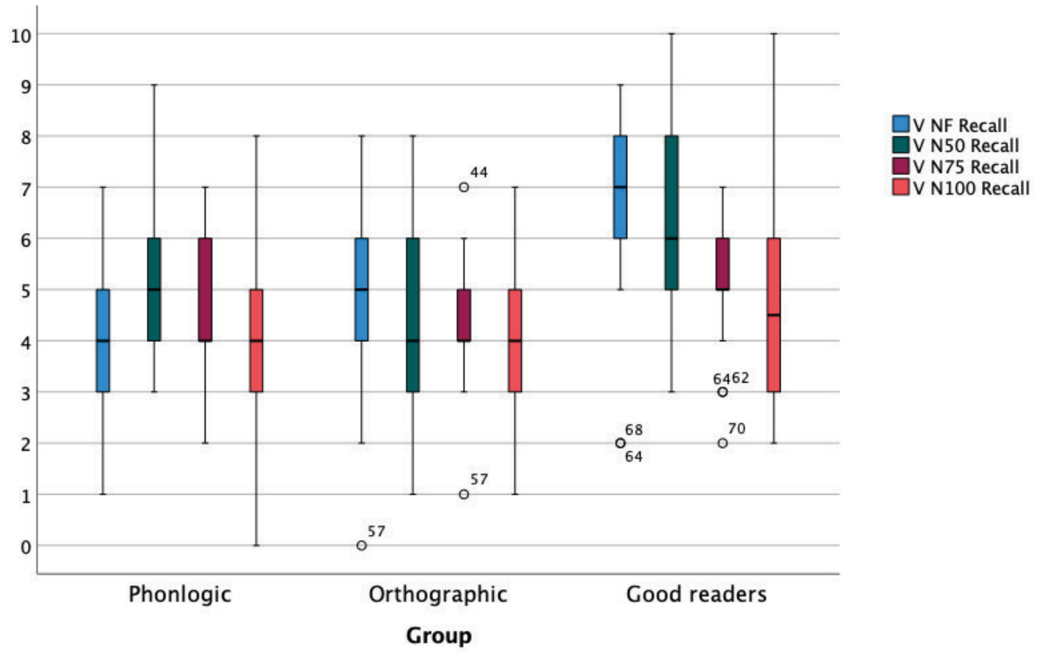

Figure 2. Boxplot showing word recall performance as a function of visual noise level
