# Supplementary material for: Sensory white noise improves reading skills and memory recall in children with reading disability
Source: Brain Behav. 2021 Jun 6;11(7):e02114. doi: 10.1002/brb3.2114 (PMC8323032; doi:10.1002/brb3.2114)
Supplement: Supplementary file 2 — Table S1‐S2 [file BRB3-11-e02114-s001.docx]

**Supplementary Tables**

**Supplementary Table 1.** Participants test scores Single Word Reading and Episodic Verbal Memory Recall tasks in the four visual white pixel noise conditions (no noise, noise 𝜎= 50, 75, 100).

| **Task /Group** | **Phonologic Group N= 30** | | **Orthographic group  N= 30** | | **Good Readers  N= 22** | |
| --- | --- | --- | --- | --- | --- | --- |
|  | M(SD) | Range | M(SD) | Range | M(SD) | Range |
| **Word Reading**  No noise  Noise 50  Noise 75  Noise 100 | 10.1 (1.7)  10.9 (1.7)  10.9 (1.5)  10.0 (2.0) | (4-12)  (3-12)  (5-12)  (3-12) | 11.4 (1.0)  11.4 (0.9)  11.3 (0.9)  11.0 (1.4) | (9-12)  (9-12)  (9-12)  (7-12) | 11.95 (0.2)  11.95 (0.2)  11.95 (0.2)  11.68 (1.3) | (11-12)  (11-12)  (11-12)  (6-12) |
| **Word recall**  No noise  Noise 50  Noise 75  Noise 100 | 4.1 (1.5)  5.1 (1.6)  4.6 (1.3)  3.8 (1.7) | (1-7)  (3-9)  (2-7)  (0-8) | 4.7 (1.7)  4.4 (1.7)  4.3 (1.2)  4.3 (1.7) | (0-8)  (1-8)  (1-7)  (1-7) | 6.6 (1.9)  6.2 (1.8)  5.2 (1.3)  4.5 (1.9) | (2-9)  (3-10)  (2-7)  (2-10) |

**Supplementary Table 2.** Participants evaluation of auditory- and visual white noise exposure during testing.

| **Group /noise type** | **Auditory noise** | | | **Visual noise** | | | **N** |
| --- | --- | --- | --- | --- | --- | --- | --- |
| **Preferred noise level** | with  noise | no noise | both  works | some noise | lots of noise | no noise |  |
| **Phonologic group** (freq) | 16 | 7 | 7 | 16 | 13 | 1 | 30 |
| **Orthographic group** (freq) | 15 | 12 | 3 | 20 | 7 | 3 | 30 |
| **Good Readers** (freq) | 8 | 7 | 7 | 17 | 3 | 2 | 22 |
| **Group comparisons** | Chi^2^= 5.21, p= ns. | | | Chi^2^= 6.56, p = ns. | | | 82 |
